# Reply 2: Birth weight as a predictor of breast cancer: a case–control study in Norway

**DOI:** 10.1038/sj.bjc.6600979

**Published:** 2003-05-27

**Authors:** L J Vatten, T I L Nilsen

**Affiliations:** 1 Department of Community Medicine and General Practice, Norwegian University of Science and Technology, Medical Research Center, N-7489 Trondheim, Norway; 2 The Norwegian Cancer Society, PO Box 5327 Majorstua, N-0304 Oslo, Norway

**Sir,**

We welcome the comments of van Noord concerning the different results for birth weight and breast cancer risk reported by ourselves (Vatten *et al*, 2002) and Sanderson *et al* (2002). He suggests that women's breast cancer risk is influenced by the preconception viability of their mothers' oocytes, particularly the quality of their mitochondria, since mitochondrial quality declines with age. Therefore, van Noord proposes that maternal age at birth is positively associated with breast cancer risk, suggesting that we reanalyse our data to test this hypothesis.

Reliable information on maternal age at birth was available in the Trondheim data, and hence this analysis is based on 186 breast cancer cases and 662 age-matched controls. We used conditional logistic regression to explore the association between the risk of breast cancer and maternal age at birth, and the estimated odds ratios are adjusted for age at first birth and parity. As shown in Table 1

**Table 1:** Odds ratios (ORs) and 95% confidence intervals (CIs) of breast cancer associated with maternal age at birth

**Variable**	**Case patients**	**Control subjects**	**OR^a^^b^**	**95% CI**
*Maternal age at birth*				
<25	56	196	1.0	Reference
25–29	60	212	1.0	0.7–1.5
30–34	41	155	1.0	0.6–1.5
⩾35	29	99	1.0	1.6–1.6
			*P* trend=0.97	

a ORs are computed using conditional logistic regression with cases and controls matched on year of birth.

b ORs are adjusted for age at first birth and parity in the regression model.

, we found no association with breast cancer risk over the four categories of maternal age at birth.

Although van Noord has proposed an interesting hypothesis, we found no evidence to support that maternal age at birth is positively associated with breast cancer risk. In light of our original findings, that both birth weight and birth length are positively associated with breast cancer risk, important mechanisms linking birth characteristics to breast cancer may be related to foetal growth. Recent research has shown that both birth weight and maternal pre-eclampsia are associated with adolescent growth and maturation (Vatten *et al*, 2003), and therefore, the intrauterine environment may initiate a tracking pattern of growth that ranges throughout childhood and adolescence. Ultimately, this may play a critical role in the development of breast cancer.

## References

[bib1] Sanderson M, Shu XO, Jin F, Dai Q, Ruan Z, Gao YT, Zheng W (2002) Weight at birth and adolescence and premenopausal breast cancer risk in a low-risk population. Br J Cancer 86: 84–88 11857016 10.1038/sj.bjc.6600009 PMC2746545

[bib2] Vatten LJ, Maehle BO, Lund Nilsen TI, Tretli S, Hsieh CC, Trichopoulos D, Stuver SO (2002) Birth weight as a predictor of breast cancer: a case–control study in Norway. Br J Cancer 86: 89–91 11857017 10.1038/sj.bjc.6600011 PMC2746526

[bib3] Vatten LJ, Romundstad PR, Holmen TL, Hsieh CC, Trichopoulos D, Stuver SO (2003) Intrauterine exposure to preeclampsia and adolescent blood pressure, body size, and age at menarche in female offspring. Obstet Gynecol 101: 529–533 12636958 10.1016/s0029-7844(02)02718-7

